# Bacterial community dynamics in lait caillé, a traditional product of spontaneous fermentation from Senegal

**DOI:** 10.1371/journal.pone.0215658

**Published:** 2019-05-10

**Authors:** Anneloes E. Groenenboom, Megan E. Parker, Anne de Vries, Suzette de Groot, Stephanie Zobrist, Kimberly Mansen, Peiman Milani, Remco Kort, Eddy J. Smid, Sijmen E. Schoustra

**Affiliations:** 1 Laboratory of Genetics, Wageningen University and Research, Wageningen, The Netherlands; 2 Laboratory of Food Microbiology, Wageningen University and Research, Wageningen, The Netherlands; 3 PATH, Seattle, Washington, United States of America; 4 Microbiology and Systems Biology, TNO, Amsterdam, The Netherlands; 5 Yoba for Life Foundation, Amsterdam, The Netherlands; 6 Department of Molecular Cell Biology, VU University Amsterdam, Amsterdam, The Netherlands; 7 ARTIS-Micropia, Amsterdam, The Netherlands; 8 Department of Food Science and Nutrition, University of Zambia, Lusaka, Zambia; Universite Paris-Sud, FRANCE

## Abstract

Spontaneously fermented food products contain a complex, natural microbial community with potential probiotic activity. The addition of a health-promoting, probiotic bacterium to these products ensures the delivery of that probiotic activity to consumers. Here, we assess the microbial community of a traditional Senegalese milk product produced by spontaneous fermentation, called lait caillé. We produced the lait caillé in a traditional way and added a probiotic starter containing *Lactobacillus rhamnosus* yoba 2012 to the traditional process. We found various species that are known for their ability to ferment milk, including species from the genera *Lactobacillus*, *Acetobacter*, *Lactococcus*, *and Streptococcus*. Our results show that the addition of *L*. *rhamnosus* to the inoculum, can result in detectable levels of this strain in the final product, ranging between 0.2 and 1 percent of the total bacterial population. Subsequent rounds of fermentation using passive back-slopping without the addition of new *L*. *rhamnosus* led to a loss of this strain from the community of fermenting bacteria. Our results suggest that the addition of probiotic strains at every fermentation cycle can enrich the existing complex communities of traditionally fermented lait caillé while traditional bacterial strains remain dominant in the bacterial communities.

## Introduction

Fermented foods are consumed worldwide and are of increasing interest to the field of public health. They include (complex) microbial communities of mainly lactic acid bacteria that are considered beneficial in stimulating a healthy gut microbiota and that promote food safety [1,2]. Most traditional fermented foods are produced by spontaneous fermentation, meaning that no starter culture is added to ferment the raw ingredients. Spontaneous fermentation results in products containing diverse microbial communities. Bacterial strains in these communities can be similar to probiotics [3]. The probiotic properties of these products may be further enhanced by enriching the microbial communities through the addition of known probiotic strains to ensure probiotic activity.

In this study, we characterized the microbial community of *lait caillé*, a fermented milk product from Senegal [4–7] using repeated rounds of traditional preparation as well as preparations including a probiotic starter culture. *Lait caillé* (“curdled milk”) is traditionally produced by the spontaneous fermentation of cow’s milk. The milk is first boiled and subsequently cooled before it is poured into a lahal—a wooden bowl that serves as the container for fermentation. Because lahals are used in succession, they contain a biofilm layer with the microbial culture from the previous fermentation cycle which then starts fermentation of the next batch.

First, we analyzed the composition and batch-to-batch dynamics of the microbial community in lait caillé. The microbial community in other spontaneously fermented milk consist of various lactic acid bacteria (LAB) in combination with other bacteria, as well as yeasts and molds [8–15]. In the current study we focussed on the bacterial composition of the final product in relation to the composition of biofilms in the lahals and the dynamics over multiple rounds of fermentation. Second, we studied the possibility of enhancing the probiotic properties of the final product, through addition of a probiotic starter culture to the initial microbial community. This starter culture, called “Yoba for Life”, was developed for the production of probiotic yogurt [1] and consists of a mixture of two bacterial strains, *Lactobacillus rhamnosus* yoba 2012 and *Streptococcus thermophilus* C106. We applied the Yoba starter culture at the start of fermentation, into lahals containing their own natural, complex microbial communities and tracked the presence of the two added strains. Additionally, in some lahals we added millet porridge, as an extra fermentable substrate for the invading probiotic bacteria, which might give them an advantage over the existing fermenting community. This laboratory-based research is complementary to the fieldwork of Parker and colleagues [5], who analyzed microbial communities of lait caillé produced in Senegal. The aim of this research was to increase the availability and accessibility of health-promoting fermented foods in regions with a high prevalence of undernutrition. Furthermore, we intended to contribute to the general knowledge on bacterial community stability, and the factors influencing bacterial invasion in existing microbial communities.

## Materials and methods

### Bacterial strains and growth media

We used bacterial invaders from the Yoba for Life starter culture for probiotic yogurt (CSK food enrichment, Leeuwarden, The Netherlands). This starter culture consists of *Lactobacillus rhamnosus* yoba 2012 and *Streptococcus thermophilus* C106 [1]. We used sachets containing one gram of dried starter culture, containing at least 1*10^9 CFU/g of each strain [16], to inoculate 1 L Ultra-high temperature (UHT) processed milk (Milbona, Lidl). MRS (Sigma-Aldrich, St. Louis, Missouri, United States) agar plates were incubated anaerobically at 28°C for the CFU count of *L*. *rhamnosus*. M17 (Fluka, Sigma-Aldrich) agar plates were incubated anaerobically at 42°C for the CFU count of the thermophile *S*. *thermophilus*.

### Fermentation in the lahals

Eight lahals were imported from different regions in Senegal (locations are reported in S1 Fig) and their biofilm was sampled upon arrival in the laboratory. From every lahal, 4 cm^2^ of the inside of each lahal was superficially scraped using a spatula. The obtained wood sample containing the microbial biofilm (inoculum for the fermentations) in these lahals was suspended in 1 mL of saline (0.8% (wt/vol) NaCl solution). The lahals were filled with 1 L UHT milk (Milbona, Lidl) and incubated at 28°C for 3 days to allow the first round of fermentation. Samples (1 mL) of the resulting fermented milk were stored at -20°C and in glycerol (0.5 mL) at -80°C. After use, the lahals were cleaned with sterile water and tissue paper. Immediately after the cleaning, a biofilm samples was taken from the inside of the lahals. The lahals were used for fermentations and regularly sampled for 4 months. MRS (Sigma-Aldrich, St. Louis, Missouri, United States) agar plates were incubated anaerobically at 28°C for the total count of lactic acid bacteria as measure for total bacterial count.

### Standard Yoba yogurt

A standard Yoba yogurt was produced by adding 1 g Yoba for Life starter culture to 1 L UHT milk in a sterile glass bottle. This bottle was incubated at 28°C overnight to allow fermentation. At different time points during fermentation samples were incubated anaerobically on M17 agar at 42°C and MRS agar at 28°C to count respectively *S*. *thermophilus* and *L*. *rhamnosus*. Resulting counts were used to calculate average growth rates in standard Yoba yogurt. The DNA was extracted from the resulting yogurt for analyses of *L*. *rhamnosus* and *S*. *thermophilus* concentrations, which were used as the standard for probiotic characteristics.

### Probiotic invasion

In the eight lahals, the following propagation environments were tested: 1) the addition of 1 g Yoba starter culture to 1 L UHT milk at fermentation cycle 1 and cycle 7 (for three lahals); 2) the addition of 1 g Yoba culture starter to 1 L UHT milk at fermentation cycle 1 and cycle 7 and a one portion of millet porridge at all twelve fermentation cycles (for three lahals); and 3) controls consisting of only 1 L UHT milk (two lahals). To produce one portion of millet porridge, 50 g millet flour was mixed with 400 mL water and heated in a microwave (750W). Heating was alternated with stirring every 30 seconds, until the porridge was boiling (typically after 4.5 minutes). The porridge was left to cool at room temperature for 30 minutes. Lahals were covered with aluminum foil and incubated at 28°C for two days and used for fermentation twelve times consecutively. Community dynamics were analyzed with non-culture based sequencing methods targeting the 16S rRNA region of the bacterial DNA.

### DNA extraction

The DNA extraction method was adapted from Ercolini et al. 2001 and Schoustra et al. 2013 [13,17]. For DNA extraction, 1 mL of fermented milk was spun down (2 minutes, 12000 RPM), after which the supernatant was removed. The cells were re-suspended in a mix of 64 μL EDTA (0.5 M), 160 μL Nucleic Lysis Solution, 5 μL RNAse, 120 μL lysozyme and 40 μL pronase E. After an incubation time of 60 minutes at 37°C and agitation of 350 RPM, 400 μL ice-cold ammonium acetate (5 M) was added and the mixture was cooled on ice for 15 minutes. The mixture was spun down and 750 μL of supernatant was transferred to a tube containing 750 μL phenol. This tube was vortexed and its content spun down (2 minutes, 12000 RPM) and 500 μL of supernatant was transferred to a tube containing 500 μL chloroform. This tube was vortexed and its content spun down (2 minutes, 12000 RPM) and 400 μL of supernatant was transferred to a tube containing 1 ml 100% ethanol and 40 μL sodium acetate (3 M). This tube containing DNA was left to precipitate at -20°C overnight. The next day, the tube was spun for 20 minutes at 12 000 RPM at 4°C. The supernatant was carefully aspirated and the DNA pellet was washed by adding 1 mL 70% ethanol. The tube was spun for 10 minutes at 12 000 RPM at 4°C, after which the supernatant was aspirated again. The DNA pellet was left to dry at room temperature and dissolved in 20 μL 10 mM Tris pH 7.5.

### DNA sequencing and sequence analyses

The extract containing DNA from all organisms in the community was sent for paired-end sequencing of the V4 hypervariable region of the bacterial 16S rRNA gene amplicon (341F-785R) on the MiSeq Illumina platform by LGC genomics (Berlin, Germany).

For further data processing and statistics the QIIME pipeline [18], modified from Bik et al. [19] was used. Paired-end reads were joined using join_paired_ends.py (with minimum overlap 10 basepairs) after which sequences were trimmed and filtered using cutadapt (v1.11 -q 20, -m 400 [20]) using the known primer sequences CCTACGGGNGGCWGCAG and GACTACHVGGGTATCTAAKCC to trim both sides of the sequence. These trimmed sequences were then checked for chimera’s, using uchime (v4.2.20, gold database [21]). Sequences with a chimera score lower than 0.28 were retained. Next, sequences were clustered into operational taxonomic units (OTUs) after quality check using pick_open_reference_otus.py (-s 0.1, -enable_rev_strand_match TRUE, -align_seqs_min_length 75, -pick_OTU_similatiry 0.95). Taxonomy of the resulting OTUs was assigned to representative sequences using the Greengenes (v13.5) rRNA database. This algorithm gives a representative sequence for an OTU, which were used to perform a local BLAST using the gold database from uchime [22]. The outcome of the top BLAST hit was used for further data processing. Shannon index (H) accounts for both abundance and evenness of OTUs present and is calculated using H = -Σ pi ln pi in which pi is the proportion of species i relative to the total number of species[23].

## Results and discussion

### Bacterial composition of lait caillé

#### Initial composition of microbial communities in lahals

**Fig 1:**
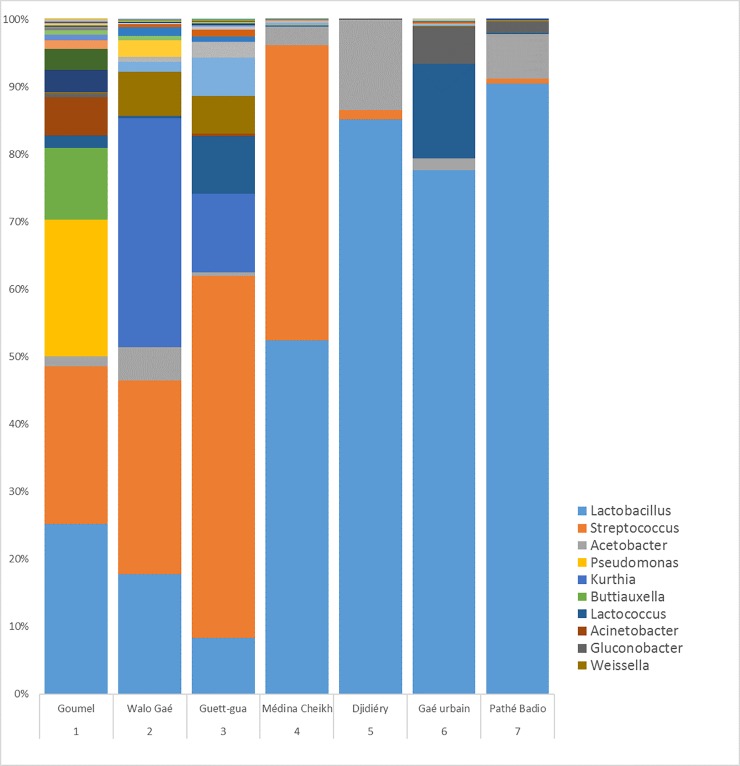
Bacterial composition of biofilm samples of all lahals upon arrival at the laboratory. Different colors indicate different genera and can consist of different species and different OTUs. The ten genera with highest abundance are indicated in the legend. The aim of the figure is to show the diversity found in the microbial communities of lait caillé in terms of genus and species variation. For specific abundances of species and OTU, please refer to the sequencing data provided with this publication. Lahal numbers are used consistently throughout the experiments. Locations indicate the origin of the lahals.

Upon arrival of the lahals in the laboratory, we analyzed the bacterial composition of their internal surface. Because the lahals are wooden, they exhibit a rough internal surface that facilitates the formation of biofilms and the accumulation of residues from in each fermentation cycle. Species of *Lactobacillus* and *Streptococcus* are the most abundant across all community structures found on the lahals’ surfaces; 56.8% and 16.8% of the total reads respectively (Fig 1). However, substantial variation between lahals was found both in terms of types of species present as well as overall species diversity. The Shannon index (H) is a measure of diversity based on operational taxonomic units (OTUs). The Shannon index varied from 1.98 to 3.20 and can be found per lahal in S1 Table. The community structures we find here are in line with the findings of Parker et al. [5], who analyzed the same type of lahals in a field study in Senegal. We found various species that are known for their ability to ferment milk, such as *Lactobacillus helveticus* [24,25] and *Streptococcus thermophilus* [26,27]. Not all species are likely to contribute to the milk fermentation. We found species that are known for their presence in the environment and animals, including *Pseudomonas libanensis* [28] and *Buttiauxella warmboldiae* [29], which have a high presence in lahal 1 (18.9% and 10.6%, respectively), and *Kurthia gibsonii* [30], which has a high presence in lahal 2 and 4 (33.9% and 11.6% respectively). Presence of species like *Lactobacillus delbrueckii* confirmed that the communities in the lahals can contain probiotic strains [31,32]. However, with the analyses used in this study, the identification of the bacteria at the level of subspecies or lineage is not possible and therefore no conclusions regarding the presence of probiotic strains can be made.

The spontaneous nature of the fermentation explains our finding that the seven lahals contain microbial communities of varying compositions. A cluster analysis did not reveal a relationship between the community structure and the origin, frequency of use, or age of the lahals (S1 Fig). However, the lahals were used by different producers, who might have different methods, which may have influenced the influx of various bacteria as well as the selection pressure on the various bacterial species.

#### Similarity between lahal biofilm community and microbial composition in the resulting lait caillé

Spontaneous fermentation of lait caillé differs slightly from the process of active back-slopping, where a sample from a previous fermentation round is used to start a new fermentation [33,34]. We therefore refer to this process as “passive back-slopping”, because an external starter culture is not intentionally added. The outcome of the first cycle of spontaneous fermentation is difficult to predict, as it is highly influenced by chance. Over time, through back-slopping, the fermenting community can stabilize and generate stable product characteristics [35,36]. In some traditional fermented dairy products, such as Parmigiano Reggiano [37], the back-slopped community must compete with a microbial community present in the raw milk. For lait caillé this is not the case, as the raw milk is boiled before fermentation. We hypothesize that the communities in the lahals are the inoculum for the milk. Thus, the microbial community in lait caillé will be a modified subset of the bacterial community in the lahals’ biofilm.

**Fig 2:**
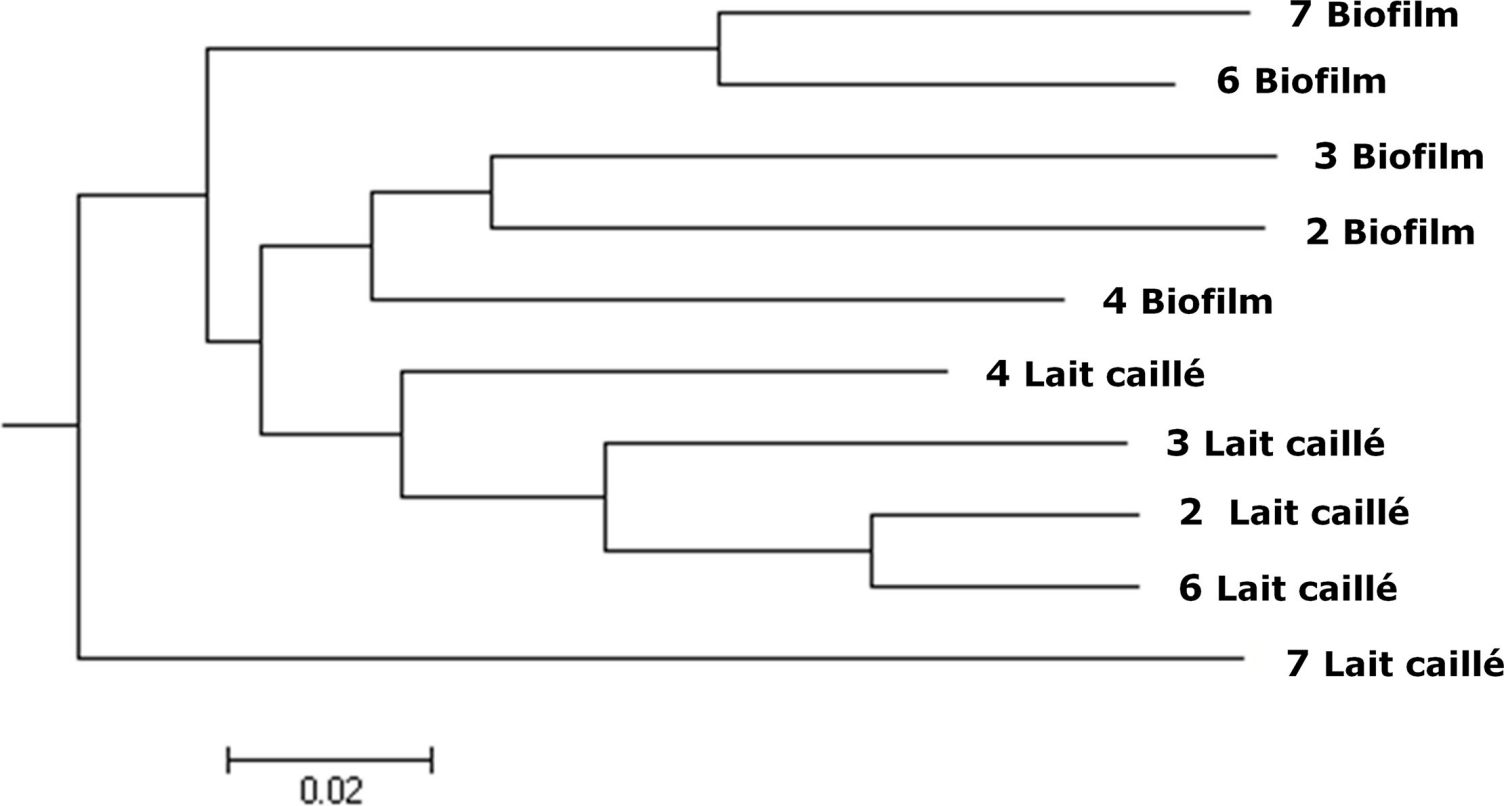
Hierarchical clustering of bacterial communities from lahal surface and lait caillé. Lahal surface samples as well as lait caillé samples of lahal 2,3,4,6, and 7 are clustered using Unweighted Pair Group Method with Arithmetic Mean based on beta-diversity of the bacterial communities.

Upon arrival at our laboratory, the lahals were used for several rounds of fermentation. Each round was initiated by adding 1 L of sterilized whole milk to each lahal, which was subsequently incubated at 28°C for 48 hours. We compared the microbial composition of lait caillé (the final product) after one fermentation cycle with the microbial composition of the biofilm in the lahal. Fig 2 shows a cluster analysis of the bacterial biofilm and final product communities of lahals 2, 3, 4, 6, and 7. This shows that biofilm samples and lait caillé samples cluster more often together than each lait caillé sample clustering with the corresponding biofilm sample. Considering that the biofilm is the only inoculum in the sterilized milk, we might expect some more overlap. However, multiple strains present in the biofilm do not grow in milk. The most abundant OTUs were different for every lait caillé sample, but for every individual lait caillé, the three most common OTUs in could be traced into the corresponding lahal biofilm samples. However, some OTUs from lait caillé are not detected in the biofilm samples. It is plausible that these bacteria were present in the biofilm at a level below the detection limit and have the chance to grow significantly when milk is added to the lahal [38].

#### Changes in the microbial composition of lait caillé over multiple rounds of fermentation

**Fig 3:**
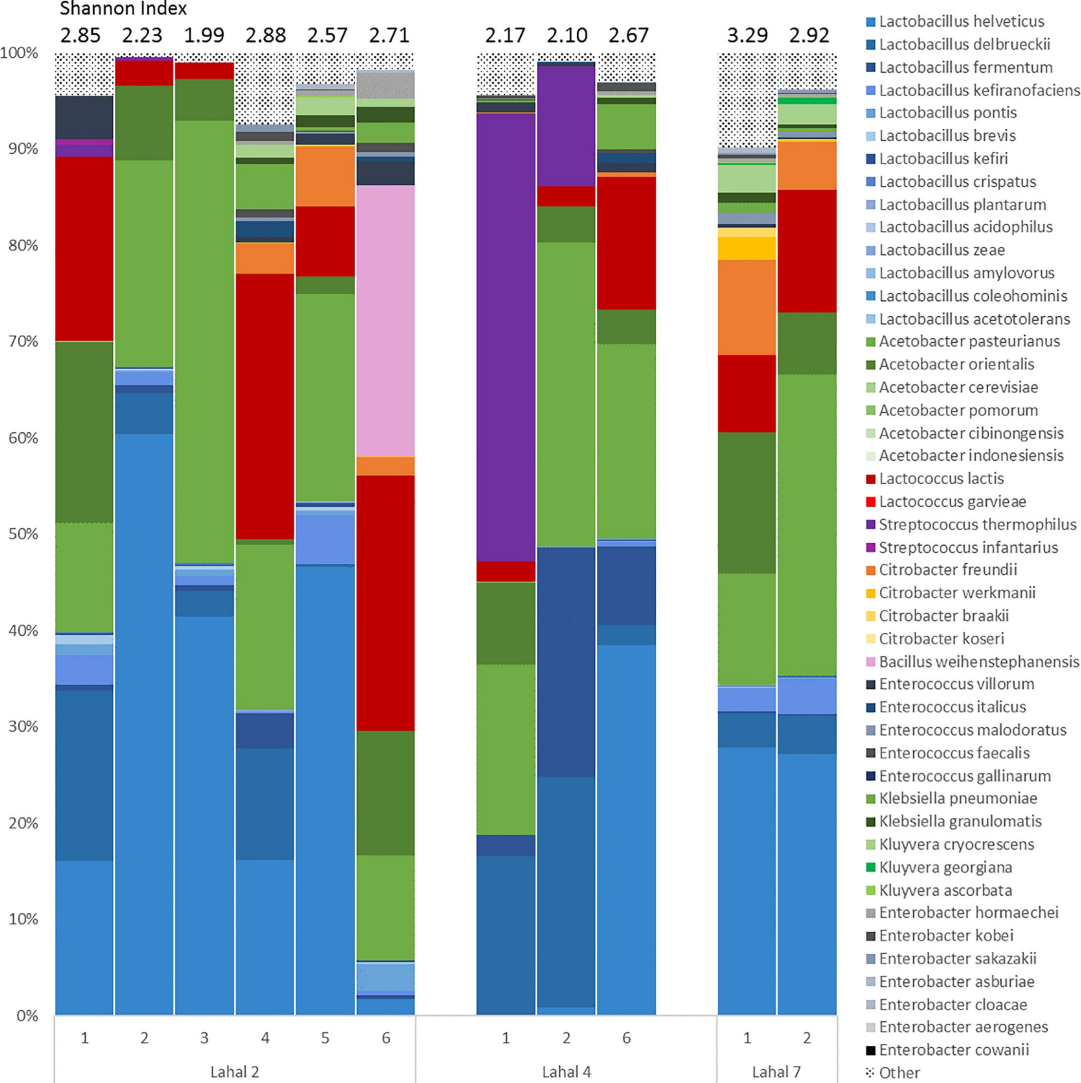
Community composition of bacterial species in lait caillé of lahals 2, 4, and 7 over time. These lahals were used for fermentation by only adding sterile milk. Data points of lahal 4 and 7 are not shown as there were other ingredients added in these fermentation. Different colors indicate different species and can consist of multiple OTUs. Vertical axes indicates percentage of total reads within the sample. The aim of the figure is to show the diversity found in the microbial communities of lait caillé in terms of genus and species variation. For specific abundances of species and OTU, please refer to the sequencing data provided with this publication.

To assess the temporal stability of the bacterial communities in the final products of three lahals, a selection experiment was conducted. These lahals were used in Senegal to propagate the communities over many cycles, allowing them to adapt to specific growth conditions. We may assume that the bacterial communities are in dynamic balance in terms of their species composition. Then, not taking erratic passersby into consideration, the bacterial frequencies will not change as much between fermentation cycles, provided that the incubation conditions in the laboratory are not very different from those in Senegal. The results (Fig 3) show that the communities of lait caillé produced in lahals 2, 4, and 7 maintain a high diversity throughout the fermentation cycles (25 transfers in case of lahal 2). Over time, we see a change in the bacteria present in the lait caillé samples. Lait caillé from both lahal 2 and lahal 9 is mainly dominated by *Lactobacillus helveticus*, *Acetobacter pasteurianus*, and *Lactococcus lactis*. Lait caillé from lahal 4 is mainly dominated by *Streptococcus thermophilus*, with *Lactobacillus delbrueckii* as the main *Lactobacillus* species after the first fermentation. After six fermentation rounds, *Lactobacillus helveticus* has the highest presence, together with *Acetobacter pasteurianus* and *Lactococcus lactis*. As this is also the main species present in lait caillé from lahals 2 and 7, these strains might form a stable community. When comparing these data to the results reported by Parker et al. [5], we see that the percentage of *Acetobacter* species is higher, while that of *Streptococcus* species is lower in these lait caillé samples. This might be due to the difference in fermentation time; each fermentation cycle in our experiment was 48 hours, compared to 12–24 in Parker et al. (6). S*treptococcus thermophilus* is known for its proteolytic activity and is therefore able to grow from the start of a fermentation [39,40]. *Acetobacter* species grow through oxidation of the fermentation products from the rest of the community [41], and will have their growth at a later stage in the fermentation. During this stage, the relative abundance of *Streptococcus* species might decrease due to a relative decrease in growth.

The difference between this selection experiment and most laboratory selection experiments using bacterial cultures (experimental evolution), is the mode of bacterial transfer between fermentation cycles [42–45]. Rather than transferring the liquid phase of the final product the lahals themselves are “transferred” to the next round of fermentation, in the sense that each lahal contains a biofilm that acts as the starter culture. This mode of transfer represents passive back-slopping, that is part of traditional lait caillé production methods. As a result, bacteria are under selection not only to grow fast in milk, but also attached to the lahals’ inner surfaces, either by formation of a biofilm, or through residing in a deep crevice in the wood of the lahal.

#### Community diversity

The bacterial diversity of the communities did not decrease throughout the fermentation cycles in the laboratory (Fig 3), which suggests that niches in the system have all been occupied in a temporally stable way. This may limit the opportunity for other bacteria such as probiotic bacteria, but also pathogens, to invade the communities. High diversity can result in a stable functionality of the community due to a back-up function [46–48], causing a lower number of unoccupied niches, for example due to unused nutrients [49]. Under such circumstances, those niches are not available for any invader, which makes the whole system more likely to keep its functionality and not destabilise due to a non-cooperator [50]. However, the exact relationship between diversity and stability is still under debate [51,52].

### Addition of Yoba starter culture

In the second part of this study, we attempted successful invasion by probiotic bacteria that were not present in the original community. If these probiotics were to become a permanent member of the bacterial community of a lahal, all products fermented in this lahal could have probiotic characteristics.

Probiotics were added in a dried form; the Yoba for Life starter culture [1], containing *Lactobacillus rhamnosus* yoba and *Streptococcus thermophilus*. This starter culture is used in Uganda and other African countries to produce Yoba yogurt, a probiotic yogurt, and has been used before in studies on nutritional enrichment of traditional foods [53]. The probiotic activity of Yoba yogurt and other foods that are enriched with the Yoba starter culture, is mainly attributed to *L*. *rhamnosus*, while *S*. *thermophilus* is necessary because of its proteolytic activity [16].

#### Presence of *L. rhamnosus* and *S. thermophilus* in traditional lait caillé

Before adding a starter culture, we screened the bacterial communities of traditional lait caillé for the presence of *L*. *rhamnosus* and *S*. *thermophilus* strains. The resolution of the 16S rRNA encoding DNA amplicon is too low to distinguish between wild *L*. *rhamnosus* strains and the *L*. *rhamnosus* yoba 2012 from the Yoba starter culture. The presence of OTUs representing *L*. *rhamnosus* and *S*. *thermophilus* in traditional lait caillé therefore has an influence on the trustworthiness of the findings in lait caillé produced with the Yoba starter culture. All lait caillé without Yoba starter culture tested negative for *L*. *rhamnosus* OTUs, while the samples from standard Yoba yoghurt and Yoba-enriched lait caillé tested positive (Table 1). This implies that the OTUs that did blast as a *L*. *rhamnosus* indeed originated from the Yoba starter culture as this strain is below detection limits in traditional lait caillé. *S*. *thermophilus* was found in some traditional lait caillé, which needs to be taken into account when analyzing bacterial communities of enriched lait caillé.

**Table 1:** Wild type strains of *L*. *rhamnosus* and *S*. *thermophilus* in lait caillé.

	Lait caillé from Lahal 3		Lait caillé from Lahal 4		Lait caillé from Lahal 6		Lait caillé from Lahal 8		Lait caillé from Lahal 9		Standard Yoba yogurt		Isolated *L. rhamnosus* Yoba	
	Avg	S.E.	Avg*	S.E.*	Avg	S.E.	Avg*	SE*	Avg	S.E.	Avg*	SE*	Avg*	SE*
***% L. rhamnosus***	0	0	0	-	0	0	0	-	0	0	0.29	-	99.73	-
***% S. thermophilus***	0.23	0.43	21.96	-	19.65	19.67	0	-	0.03	0.03	99.26	-	0	-
***# OTUs L. rhamnosus***	0	0	0	-	0	0	0	-	0	0	3	-	99	-
***# OTUs S. thermophilus***	1	1	26	-	46	38	0	-	1	1	286	-	0	-

Percentages and total numbers of OTUs found in lait caillé before the addition of Yoba starter culture, standard Yoba yogurt, and Lactobacillus rhamnosus Yoba isolated from the standard Yoba yogurt that blasts as *L*. *rhamnosus* or *S*. *thermophilus*.

*values based on one measurement.

#### Yoba starter culture concentration after systematic addition of invaders

As the next step, we performed an invasion experiment to assess if the concentration of Yoba bacteria in lait caillé can become as high as in standard Yoba yogurt, in this way reaching levels for the product to be considered probiotic. Standard Yoba yogurt has a bacterial concentration of 10^6^ to 10^7^ CFU/mL, and contains mostly *S*. *thermophilus* with approximately 0.3% *L*. *rhamnosus*. Because most of the bacterial communities found in the lahals are relatively diverse, invasions into these communities will likely be difficult. To mitigate this, we added the invading bacteria to the community in very high amounts, so the invaders initially outnumber the biofilm-inhabiting bacteria by a factor of 10. We measured 6.36±2.47 x 10^5^ CFU/mL bacteria released from the biofilm 10 minutes after the start of fermentation and 6.61±0.13 x 10^6^ CFU/mL invading bacteria. A higher starting frequency of invaders improves their chances of integrating into the community before being outcompeted for nutrients, and increases their odds of outcompeting others by drift processes and chance.

**Fig 4:**
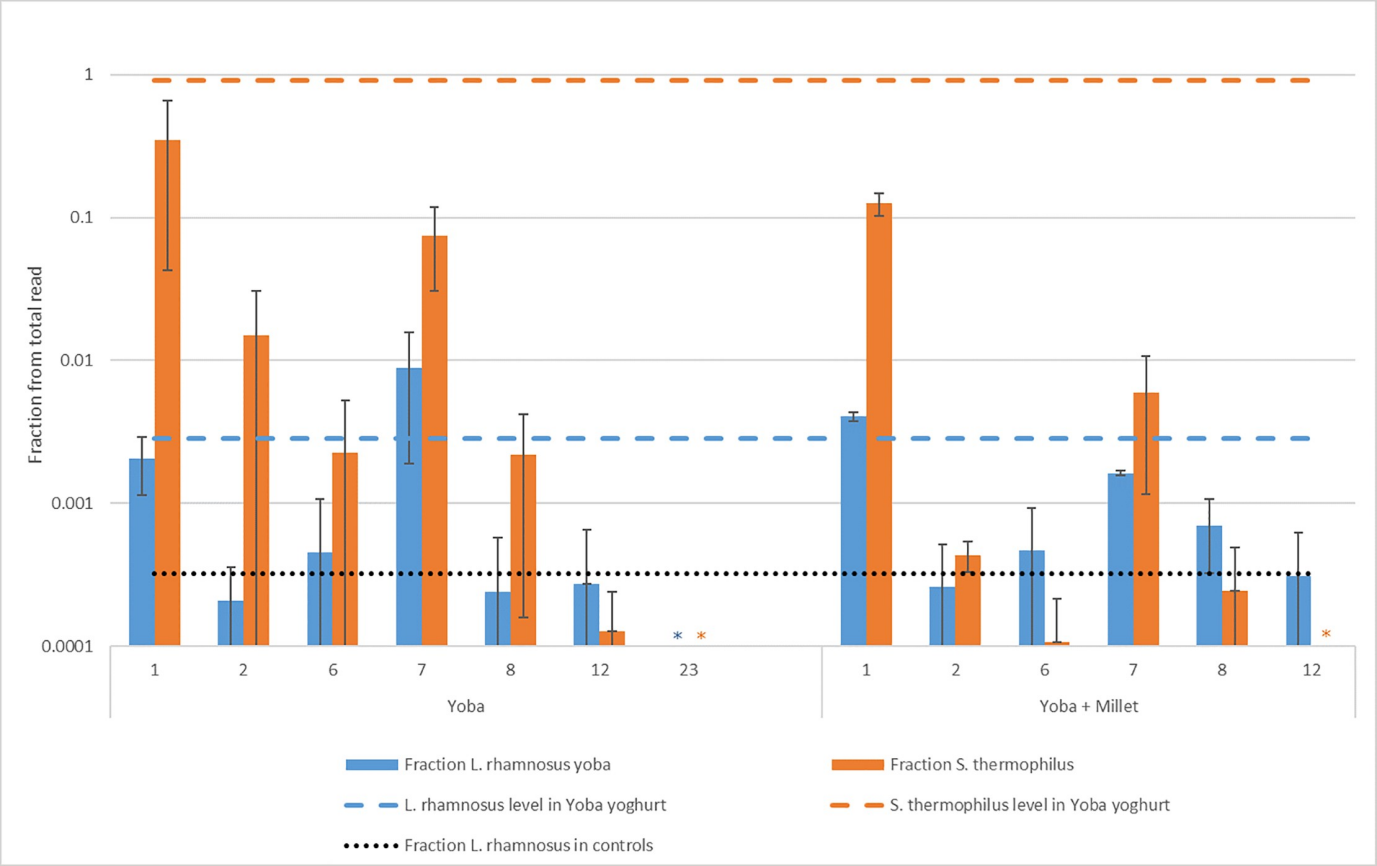
Fraction of *S*. *thermophilus* and *L*. *rhamnosus* in total microbial community in lait caillé. Yoba: Yoba starter culture addition in lahal 3, 6 and 7 at time points 1 and 7. Millet: Yoba starter culture and millet porridge addition in lahal 2 and 8 at time points 1 and 7 and addition of millet porridge at all 12 time points. The striped lines show concentration of *S*. *thermophilus* (orange) and *L rhamnosus* Yoba (blue) in the Yoba yogurt (produced in standard sterile conditions). The dotted line shows *L*. *rhamnosus* Yoba levels found in controls. * indicates samples where invader levels were below the detection limit. Standard error is indicated with error bars.

After the addition of the Yoba starter culture at the start of the first and seventh fermentation cycle, we performed multiple rounds of fermentation in the same lahals without the addition of Yoba starter culture. In this way, we assessed the success of establishing the probiotic starter bacteria in the lahals’ biofilms, which serves as the inoculum for all following lait caillé fermentations in that lahal. The presence of invading strains was measured after completion of the fermentation cycle during which they were added (cycles 1 and 7), as well as some of the subsequent fermentation cycles (cycles 2 and 6; 8 and 12, see Fig 4). The average percentage of *L*. *rhamnosus* in Yoba yogurt was used as a threshold baseline value to classify the Yoba-enhanced lait caillé as probiotic.

In fermentations where Yoba starter culture had been added, the two Yoba species made up between 4 and 80 percent of the total microbial population after 48 hours. In these fermentation cycles (cycles 1 and 7), *L*. *rhamnosus* reached concentrations similar to those found in standard Yoba yogurt (blue dashed line, Fig 4), while the concentrations of *S*. *thermophilus* stayed below the standard (orange dashed line). In the fermentation cycles that followed (cycles 2, 6, 8, and 12), the abundance of both *L*. *rhamnosus* and *S*. *thermophilus* had dropped to the level of the negative control lahals (black dotted line). Lahal 6 underwent 23 fermentation cycles. At this point, both *L*. *rhamnosus* and *S*. *thermophilus* concentrations were below the detection limit (indicated by * in Fig 4).

In order to provide the invading bacteria an advantage over the existing community in lait caillé, another fermentable substrate, cooked millet porridge, was added at the beginning of every cycle in two lahals. Both *L*. *rhamnosus* and *S*. *thermophilus* are known to grow well when provided with millet porridge [54]. The bacteria in the lahal are not adapted to millet as a substrate. When the community encounters a disturbance, this might open up the possibility for an invader to take a place in the community [50,55]. Addition of millet porridge is common practice in lait caillé production in Senegal, although it is usually added prior to consumption rather than at the start of fermentation. In Fig 4, we see that the addition of millet porridge does not seem to affect the invasion by these bacteria. The concentrations either of the two species from the Yoba starter culture are comparable to the invasion without addition of millet porridge. Further research could investigate other nutrient sources which provide a more selective environment that benefits the added strains.

In the work of Parker and colleagues [5], the addition of Yoba starter culture to various lahals with lait caillé resulted in a 20- to 60-fold increase in the total number of probiotic bacteria in the lait caillé. These findings are in line with ours. Upon the addition of Yoba starter culture, we found a concentration of *L*. *rhamnosus* strains similar to the concentration found in standard Yoba yogurt. However, the invading strains were unable to successfully establish themselves in the lahals in order to classify the lait caillé produced in the following fermentation rounds as a probiotic product. *L*. *rhamnosus* was found in some biofilm samples, but in very low concentrations (results not shown). It is therefore unlikely that this strain established itself in the biofilm to provide a *L*. *rhamnosus* inoculum for the next batch of lait caillé. When adding Yoba to a Zimbabwean milk product called mutandabota, Mpofu et al. [53] found that the concentration of *L*. *rhamnosus* yoba at the end of fermentation was 8.8 log CFU/ml, a considerably higher concentration than found in all samples of lait caillé. An important difference between lait caillé and mutandabota is that in the latter no original community is present with which the added strains would have to compete. Further research could include a selection step, to obtain probiotic bacteria that have the ability to form biofilms.

#### Influence of Yoba starter on bacterial composition of lait caillé

**Fig 5:**
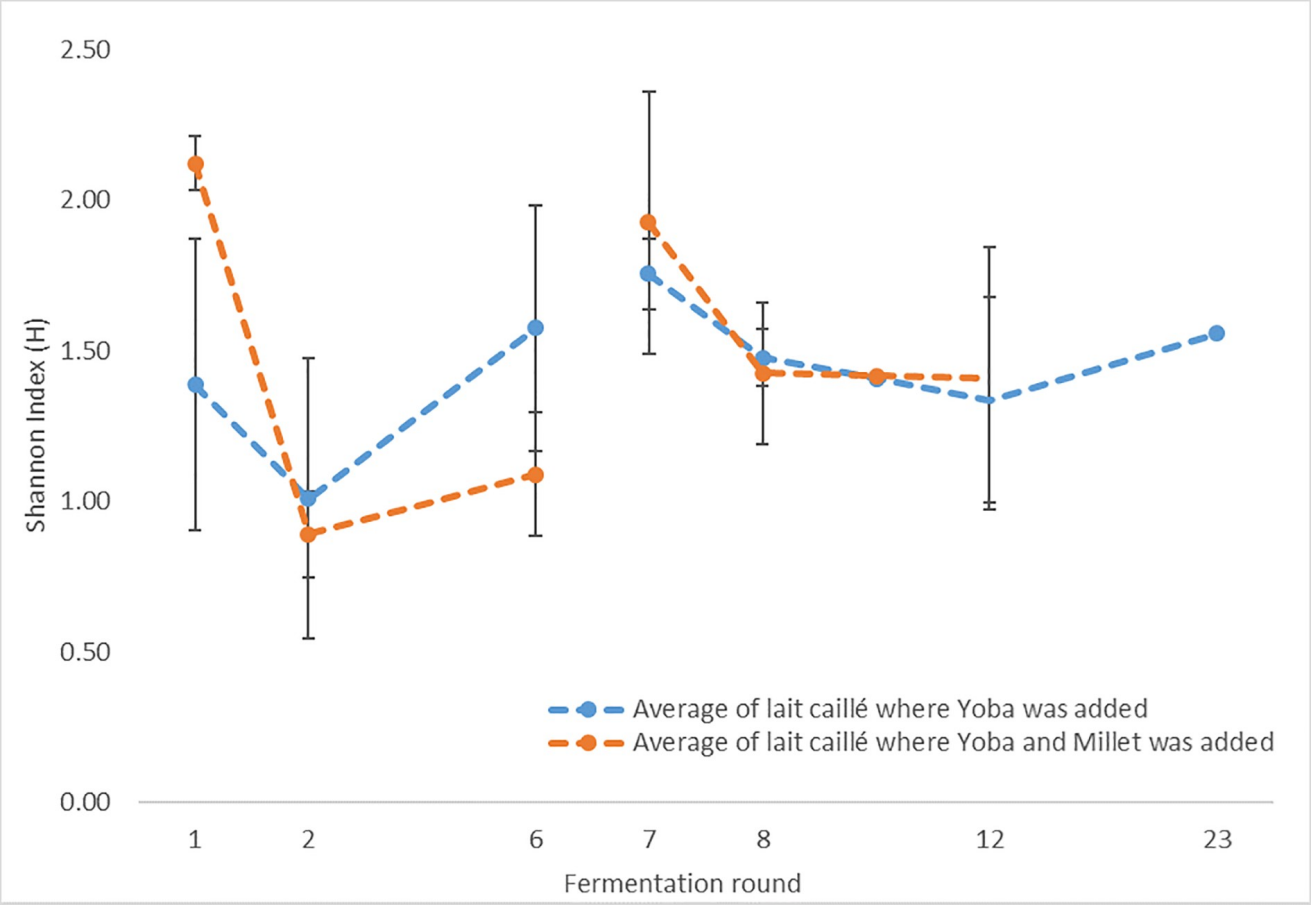
Microbial diversity in lait caillé during and after the addition of the Yoba starter culture. Average Shannon index of lahals inoculated with Yoba (lahals 3, 6, and 7) and lahals inoculated with Yoba starter culture in combination with millet porridge (lahals 2 and 8). Error bars indicate standard deviation. Yoba starter culture is added to all lahals at fermentation round 1 and 7, millet was added to lahals 2 and 8 at all fermentation rounds (fermentations rounds 3,4,5,9,10,11, and 13 up to 22 were not measured).

After the invasion experiment, the community structures of these five lahals were analyzed. There was no significant change in the Shannon index following the Yoba culture addition with or without millet porridge (Fig 5). Nevertheless, we see a trend of increased bacterial diversity as a result of the addition of Yoba starter culture. In the lahals where millet was added along with the Yoba starter culture, this effect seems to be greater. Possibly some strains benefit from the disturbance caused by the invaders and the addition of millet which can lead to greater diversity.

**Fig 6:**
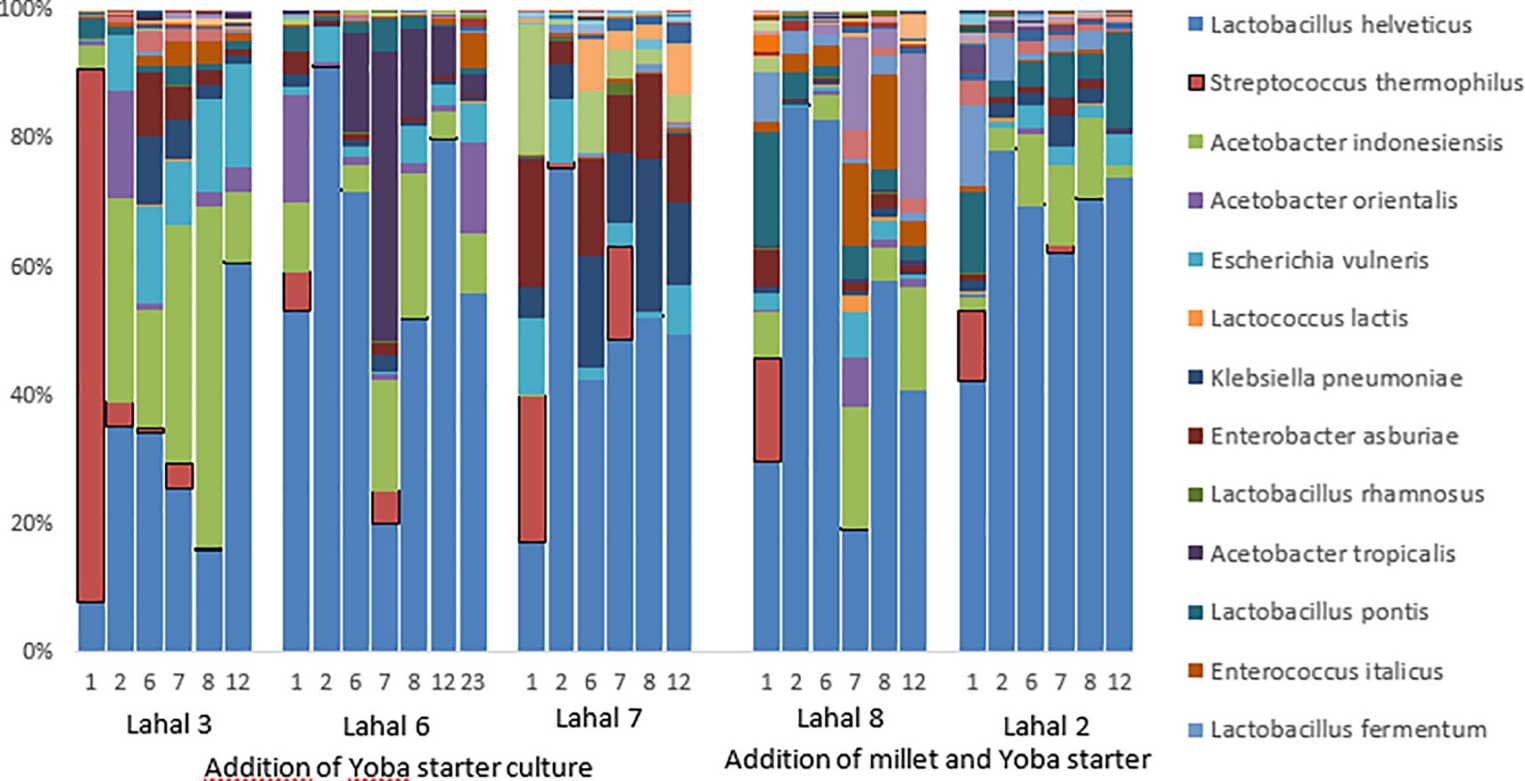
Community composition of the bacterial species in lait caillé of lahal 2, 3, 6, 7, and 8 during the invasion experiment with Yoba starter culture. Numbers indicate fermentation rounds. Vertical axes indicates percentage of total reads within the sample. The thirteen species with highest abundance are indicated in the legend. The aim of the figure is to show the diversity found in the microbial communities of lait caillé in terms of genus and species variation. For specific abundances of species and OTU, please refer to the sequencing data provided with this publication.

Upon the addition of invaders, the community structure of a lahal can change (Fig 6). The increase in one species can cause the abundance of another species to change [56]. Because the lahals all contain a different microbial composition in the biofilm, also the effects of the Yoba starter culture can be different. As expected, we see an increase in *S*. *thermophilus* in fermentation rounds 1 and 7. In addition to a diversity change, in lahals 6 and 8 we observe a decrease in *L*. *helveticus*. Without the addition of the Yoba starter culture, this strain has the highest abundance. Besides this change, the addition of Yoba starter culture seems to influence the presence of *Acetobacter* species. In lahal 6 the addition was followed by an increase in *Acetobacter orientalis* and *Acetobacter tropicalis*. As the results shown are all relative abundances, we cannot conclude whether the increase or decrease of a certain species was also absolute. Comparing the community structures of lahals before and after the second addition of Yoba starter culture at fermentation cycle 7 reveals that the specific bacterial community of these lahals does not change greatly upon the addition of Yoba starter culture. Aroma composition analysis and other sensory analyses can be carried out to determine whether this translates to stable product characteristics.

Overall, both strains from the Yoba starter culture could grow in the lahals and remained present in the resulting lait caillé. The values of *L*. *rhamnosus* Yoba in lait caillé are similar to those found in standard Yoba yogurt. The values of *S*. *thermophilus* are lower than in standard Yoba yogurt. As other bacteria in the traditional lait caillé community can perform the proteolytic activity, the presence of *S*. *thermophilus* might not be essential.

The strains in the Yoba starter culture were not able to become members of the lait caillé community remaining in the lahals and were unable to successfully invade the biofilms. The strains might not be able to become part of any biofilm, due to their own characteristics pertaining to biofilm formation; and/or they were not able to become part of these specific biofilms due to a combination of competition with other bacteria and environmental factors. The diversity found in the lahal communities is very high. This decreases the possibility that an invader will find an unoccupied niche where no competition is present, or a niche in which it is better adapted than the original inhabitant. To successfully invade, the invader either must take over the niche of another player in the community or break up the stability of the community by interfering with existing stable interactions. For the added strains their niche in the biofilm is unknown. Species of the *Lactobacillus* and *Streptococcus* genera are present in the biofilm, which suggests the possibility for the added Yoba starter strains to become part of the community. However, the strains found in the lahals most likely have had many generations in that environment giving them the opportunity to optimally adapt to their environment. Furthermore, the growth rate of the starter culture is much lower than the growth rate of the original bacterial community in the lahals. Although we added the Yoba starter culture in very high amounts, the time the starter strains were given to adjust to the environment might have been insufficient to for them to outcompete the traditional community. The chance of successful invasion might be increased by selection of *Lactobacillus rhamnosus* Yoba that have gained the ability to form a biofilm without losing their probiotic characteristics due to genome instability [57]. Another solution can be sought in back-slopping higher amounts of lait caillé produced with Yoba starter culture or standard Yoba yogurt. Alternatively, probiotic strains originating from the natural community of lait caillé could be characterised, isolated and used to enhance the probiotic properties.

### Scientific relevance

We studied bacterial community dynamics inside wooden lahals over time to observe the naturally occurring variations in community composition. In batch fermentations the fermenting bacteria undergo nutrient cycles. Nutrient levels decrease from a high level while the environment becomes more harsh, due to a lowering of the pH. Nutrient cycling occurs often in nature, for example due to tidal or seasonal changes or in predator-prey interactions. As research on community dynamics is often performed using synthetic communities [58], adding an element of natural variation can give further insight into bacterial population dynamics. In this study, we demonstrate that natural variation is maintained in lait caillé over several fermentation rounds, while no bacteria are added to the lahal. The lahal therefore must contain the inoculum for the lait caillé, even though the microbial communities found in the lahals do not show great similarities between to the microbial communities of the lait caillé they produce.

Aside from nutritional benefits, the addition of a probiotic strain to an existing natural community can give insight into bacterial community stability and resilience. Spontaneously fermented milk products can serve as a model system for research into microbial ecology and evolutionary adaptation. In this study, we see that the initial Shannon index is not a good predictor of the success of invasion of the Yoba starter culture strains. This is not in line with the expectation that a higher initial microbial diversity comes with fewer unoccupied niches and therefore a lower chance of successful invasion into the community [49]. Most likely, natural communities have such diverse structures that the Shannon diversity alone is not an accurate indication for invasion success. Other factors, such as the presence of certain bacterial strains or interaction networks between species, might be more relevant indicators. Stability of the microbial community might result in the stability of product characteristics like taste, nutritional composition, and safety.

### Societal relevance

Lait caillé can successfully be enhanced to a probiotic product when Yoba starter culture is added at the start of every fermentation cycle. The addition of Yoba starter culture can result in a product with different organoleptic properties and may have positive effects on the health of consumers in Senegal [1]. Probiotic strains can positively influence the composition of the gut microbiota, leading to diverse health benefits, especially for vulnerable groups[59]. In regions with widespread undernutrition, increased consumption of traditional fermented food products can provide an opportunity to improve health.

The lahals used in this study are very important for the households or families that owns them. Typically, lahals are a wedding gift and provide a household with a unique, personalised lait caillé. Due to this, using traditional lait caillé to enhance nutritional standards is valuable as well as risky. It is valuable because this product is widely consumed and accepted, especially compared to other probiotic products. Other products may be too expensive and/or not well accepted by consumers [14]. In contrast, enhancement is also risky because the producers could fear unwanted changes in the product characteristics and be unwilling to change their production method.

To protect the original organoleptic properties of the lait caillé, changes to the microbial community should be kept to a minimum. Other important attributes to local fortified products are safety, cost, and ease of local production. Due to the potential health benefits of probiotic enhanced lait caillé, producers of the enhanced lait caillé might also benefit economically. However, the addition of probiotic strains will change production methods and may increase costs. The addition of a starter culture in every fermentation cycle might not be feasible for a sustainable business model in low- and middle-income countries. Ideally, this probiotic bacterium should be able to survive and proliferate in the natural microbial community. Spontaneous fermentation has lower costs and can result in product characteristics that cannot be attained by fermentation with one or two strains in a starter culture [60].

### Limitations

Bacterial communities in the lahals are unique. This causes the reproducibility of the experiments performed in this project to be relatively low. To have the ability to perform the experiments in duplicates, pieces of the same lahal which include a biofilm could be distributed over different sterile fermentation vessels. In this way inoculating various fermentations with the same starter.

The DNA analyses method used was only able to detect a wide variety of bacterial DNA. However, it is very likely the microbial community consists of different classes of microorganisms such as yeasts and viruses. Also, this method does not in all cases allow determination of bacteria to a subspecies level. Other techniques, such as mRNA analyses and PCR techniques using specific primers for target organisms, such as lactic acid bacteria, could be used to get a broader view of the total community present in the lahals and the lait caillé.

## Conclusion

In conclusion, we found that lait caillé harbors a diverse bacterial community of lactic acid bacteria that is stable in terms of composition over multiple rounds of fermentation. Although the biofilm of the lahals is the only inoculum for the lait caillé produced in this study, the bacterial composition of lait caillé differs from that in the biofilms as some strain in the biofilm grow much better in milk than others. The addition of Yoba starter culture to every fermentation cycle of a spontaneously fermented milk product like lait caillé can result in a probiotic product. It is remarkable that even a diverse community such as the bacterial community found in the lahals allows for the temporary inclusion of another species. This knowledge can inform further research aimed at leveraging the benefits of other traditional and widely consumed products supplemented with Yoba starter culture. Future research on traditionally-produced lait caillé may generate evidence of probiotic properties of the microbiota in it and hence of lait caillé itself.

## Supporting information

S1 Table Community structure of wall scraping of lahals upon arrival in the laboratory. (DOCX)

S1 Fig Available information about the lahals. Details of the origin and previous use of lahal 1 to 7. Hierarchical cluster tree is based on OTUs of the biofilm samples of the lahals.
(TIF)
