# Supplementary material for: Bacterial community dynamics in lait caillé, a traditional product of spontaneous fermentation from Senegal
Source: PLoS One. 2019 May 10;14(5):e0215658. doi: 10.1371/journal.pone.0215658 (PMC6510411; doi:10.1371/journal.pone.0215658)
Supplement: S1 Table — (DOCX) [file pone.0215658.s001.docx]

**S1 Table. Community structure of wall scraping of lahals upon arrival in the laboratory.**

| lahal | original |  | Blast | | Shannon index | | |
| --- | --- | --- | --- | --- | --- | --- | --- |
|  |  | #OTUs | #Species | #Genera | OTUs | Species | Genera |
| 1 | 2 | 237 | 87 | 47 | 3.20 | 2.57 | 2.06 |
| 2 | 3 | 122 | 43 | 30 | 3.05 | 2.20 | 1.74 |
| 3 | 4 | 112 | 49 | 35 | 2.83 | 2.06 | 1.66 |
| 4 | 6 | 70 | 28 | 18 | 1.98 | 1.20 | 0.87 |
| 5 | 7 | 65 | 18 | 7 | 2.03 | 1.19 | 0.47 |
| 6 | 8 | 73 | 27 | 18 | 2.24 | 1.22 | 0.78 |
| 7 | 9 | 90 | 29 | 16 | 2.35 | 1.27 | 0.42 |
| 8 | 0 | New lahal, not previously used | | | | | |
