# Supplementary figures and images for: Bacterial community dynamics in lait caillé, a traditional product of spontaneous fermentation from Senegal
Source: PLoS One. 2019 May 10;14(5):e0215658. doi: 10.1371/journal.pone.0215658 (PMC6510411; doi:10.1371/journal.pone.0215658)

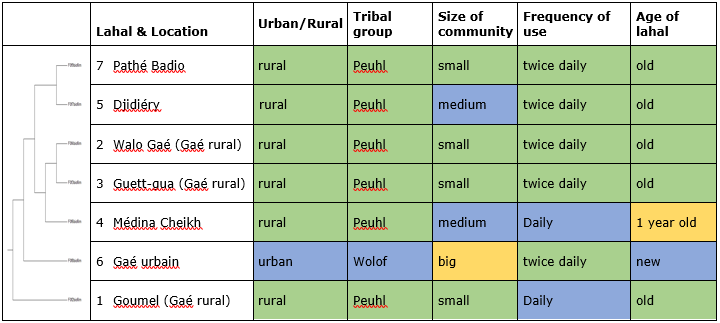

Supplement: S1 Fig — Details of the origin and previous use of lahal 1 to 7. Hierarchical cluster tree is based on OTUs of the biofilm samples of the lahals. (TIF) [file pone.0215658.s002.tif]
